# Slaying the Serpent: A Research Agenda to Expand Intervention Development and Accelerate Guinea Worm Eradication Efforts

**DOI:** 10.4269/ajtmh.23-0889

**Published:** 2024-07-09

**Authors:** Maryann G. Delea, Alexandra Sack, Obiora A. Eneanya, Elizabeth Thiele, Sharon L. Roy, Dieudonne Sankara, Kashef Ijaz, Donald R. Hopkins, Adam J. Weiss

**Affiliations:** ^1^ Guinea Worm Eradication Program, The Carter Center, Atlanta, Georgia;; ^2^ Vassar College, Poughkeepsie, New York;; ^3^ Parasitic Diseases Branch, Division of Parasitic Diseases and Malaria, U.S. Centers for Disease Control and Prevention, Atlanta, Georgia;; ^4^ Department of Control of Neglected Tropical Diseases, World Health Organization, Geneva, Switzerland

## Abstract

Dracunculiasis, also known as Guinea worm disease, is targeted to become the second human disease and first parasitic infection to be eradicated. The global Guinea Worm Eradication Program (GWEP), through community-based interventions, reduced the burden of disease from an estimated 3.5 million cases per year in 1986 to only 13 human cases in 2022. Despite progress, in 2012 Guinea worm disease was detected in domesticated dogs and later in domesticated cats and baboons. Without previous development of any Guinea worm therapeutics, diagnostic tests to detect pre-patent Guinea worm infection, or environmental surveillance tools, the emergence of Guinea worm disease in animal hosts—a threat to eradication—motivated an assessment of evidence gaps and research opportunities. This gap analysis informed the refinement of a robust research agenda intended to generate new evidence and identify additional tools for national GWEPs and to better align the global GWEP with a 2030 Guinea worm eradication certification target. This paper outlines the rationale for the development and expansion of the global GWEP Research Agenda and summarizes the results of the gap analysis that was conducted to identify Guinea worm–related research needs and opportunities. We describe five work streams informed by the research gap analysis that underpin the GWEP Research Agenda and address eradication endgame challenges through the employment of a systems-informed One Health approach. We also discuss the infrastructure in place to disseminate new evidence and monitor research results as well as plans for the continual review of evidence and research priorities.

## INTRODUCTION

Dracunculiasis, also known as Guinea worm disease (GWD), is caused by the parasitic nematode *Dracunculus medinensis*.^1^ Guinea worm disease is an ancient malady known to cause considerable physical pain and potentially substantial economic impacts in endemic areas. Guinea worm disease is recognized as a neglected tropical disease that mostly affects populations with limited access to improved drinking water in low-income countries.^2^^,^^3^ In 1986, when the World Health Assembly declared global elimination (i.e., eradication) of GWD as a goal,^4^ the disease affected an estimated 3.5 million people in 20 countries annually.^5^ Global eradication efforts over the last 40 years have eliminated transmission of the parasite from 16 affected countries and certified 200 countries free of the disease, placing the pathogen close to extinction.^6^

There are currently no vaccines or therapeutic interventions for GWD, and there are no existing early diagnostic tests to detect pre-patent infection.^7^ There are, however, multiple simple and effective interventions available that are mostly behavioral in nature and are designed to intervene upon various points of the Guinea worm (GW) life cycle.^8^ Given that existing interventions facilitated a 99.9% reduction in observed GWD from 1986 to 2010^9^ and appeared to reaffirm prior understanding of the parasite’s natural history, there was little perceived need for research into the parasite’s biology and development of additional therapeutics or early diagnostic interventions to combat GWD. Consequently, the criteria for certification did not require implementing diagnostic tools to detect pre-patent infection. However, recent changes in the relative incidence of infection among definitive nonhuman host species and related implications for the broader understanding of GW transmission pathways and dynamics have necessitated a re-evaluation of the need for a more robust Guinea Worm Eradication Program (GWEP) Research Agenda.

Prior to 2012, it was believed that GWD largely affected humans as its most common definitive host. However, disease detection among domesticated dogs (*Canis familiaris*) in Chad in 2012^10^ and laboratory confirmation of *D. medinensis* as the causative disease agent of the animal infections substantiated occasional historical accounts that animals also serve as definitive hosts for GWD.^1^^,^^11^ Laboratory-confirmed GWD has since been detected among domesticated dogs (Chad, Ethiopia, Mali, Angola, South Sudan, Cameroon) and cats (*Felis catus*; Chad, Ethiopia, Mali, Cameroon), African wild cats (*Felis lybica*; Chad), and Olive baboons (*Papio anubis*; Ethiopia).^6^^,^^12^ Since 2015, annual GWD counts among nonhuman animal hosts have consistently comprised a higher proportion of the global burden of GWD than human case counts.^13^

### Eradication endgame challenges: Impetus for an extensive, rigorous GWEP Research Agenda.

Ongoing GW transmission among human and animal hosts in the few remaining endemic countries threatens the goal of GWD eradication by 2030, as outlined in the Roadmap for Neglected Tropical Disease 2021–2030,^14^ and has extended this human health problem into a One Health paradigm. As a result, the WHO and the International Commission for the Certification of Dracunculiasis Eradication have updated the guidelines for certifying the absence of *D. medinensis* transmission to include providing evidence of parasite absence among animal hosts.^15^^,^^16^

The detection of GWD in animals motivates additional research to better understand all pathways of infection and to develop additional tools and resources for national GWEPs to accelerate their efforts, provide evidence for certification, and better align the global GWEP with the 2030 eradication target. Novel research on GW’s multiple-host pathogen system^17^ (i.e., the system that involves infection of multiple host species with GW, including an obligate intermediate host, facultative transport or paratenic hosts, and numerous mammalian definitive hosts) is intended to achieve the following objectives:

1. Refine and expand existing knowledge of *D. medinensis* parasite biology and population dynamics;
2. Help clarify intermediate, paratenic, and transport host biology, behavior, and ecology;
3. Further elucidate disease transmission among and between definitive hosts;
4. Detect GW larvae at various developmental stages (e.g., L1, L3) in environmental media, both in and out of the intermediate copepod host, to facilitate environmental surveillance;
5. Diagnose GW infection early, before the onset of prodromal signs/symptoms and patent infection;
6. Develop a therapeutic to prevent and treat GWD among definitive animal hosts; and
7. Identify effective intervention implementation strategies to promote the systematic uptake of research findings and to improve intervention uptake more broadly.

Research efforts applying technologies and methodologies that were unavailable or unknown at the inception of the global GW eradication campaign may help identify existing tools that could be repurposed and deployed to address GW and/or inform the design of novel or enhanced evidence-based interventions, which may further exploit vulnerabilities along the GW life cycle as well as the selective pressures acting upon them (i.e., biological choke points^18^). Such tools would also provide evidence of the absence of GW necessary for achieving certification. Additional implementation research initiatives are also warranted to accelerate efforts to bridge the gap between research, policy, and practice to improve intervention uptake and adoption of GW-preventive behaviors and practices as well as downstream GWD outcomes.^19^^,^^20^ It is now widely accepted that a multipronged, multidisciplinary One Health approach is needed to successfully address this stage of the GW campaign, which has clear environmental, animal, and human health components.^21^^,^^22^

The purpose of this paper is to provide a rationale for and details related to the development and expansion of the GWEP Research Agenda, which is specifically designed to address eradication endgame challenges and facilitate progress toward and achievement of GWD eradication through the employment of a systems-informed One Health approach. Systems-informed approaches consider the system’s complexities (including its underlying structures, interconnections, and dynamics) when designing solutions, and One Health approaches acknowledge the convergence of human health, animal health, and environmental health. This paper summarizes the results of a GW research gap analysis that was conducted to identify evidence gaps and research opportunities and describes the five work streams that underpin the GWEP Research Agenda. Each work stream’s scope of work within the larger GWEP Research Agenda is summarized, as are work stream–specific progress and evidence to date.

## MATERIALS AND METHODS

A GW research gap analysis, informed by agenda-setting efforts of other disease eradication programs,^23^ was conducted in 2020 to identify evidence gaps and research opportunities and to prioritize related research initiatives to achieve zero incidence of GWD by 2027, for certification of GWD eradication by 2030. The purpose of the comprehensive gap analysis was 3-fold: 1) to build a culture of inclusiveness and ownership among all GWEP researchers and stakeholders; 2) to set a comprehensive foundation for potential research areas that could perhaps be leveraged with new partners and their ongoing research; and 3) to establish a formal program reference document to map out the research agenda.

Prior to the formal GW research gap analysis, input was solicited from GWEP stakeholders (i.e., program managers, staff, researchers, and partners) to inform the final design and execution of the analysis. Stakeholders were asked to outline the gaps they observed in ongoing and past GW research activities. This feedback was complemented by additional inputs from external public health and animal health experts who had not previously conducted research on GW to ensure that potential unknown gaps were not overlooked.

The formal execution of the GW research gap analysis involved the review of available evidence about each stage of the GW life cycle. Focus was placed on highlighting understudied issues that, if addressed, could markedly decrease parasite loads, as these represent inherent biological choke points and provide the most promise for targeted research on potentially effective interventions. Preliminary stakeholder input, including from previous research convenings, identified thematic areas requiring investigation. Evidence gaps in each targeted thematic area were highlighted by contrasting current knowledge and prominent unknowns, vulnerabilities, and research opportunities with the global GWEP’s vision for the period from 2020 to 2027. Research initiatives that demonstrated work underway in 2020 and suggestions from internal and external GWEP research partners and stakeholders for additional initiatives to bridge these gaps were considered when drafting the initial iteration of the proposed scope of work for the GWEP Research Agenda. The GW research gap analysis highlighted numerous basic and applied research gaps, some of which would not be feasible for execution, given the timelines for research relative to the timeline for eradication if initiated de novo.

In the first iterations of the GW research gap analysis, evidence and research gaps were organized into five thematic areas: parasite and copepod biology, animal hosts, behavior change, diagnostics, and therapeutics. A subsequent review of the GWEP Research Agenda was conducted during June–August 2023, and additional evidence gaps and research opportunities were added. As part of that review, the five original thematic areas were re-envisioned to better align with GW’s multiple-host pathogen system and to formalize five GWEP Research Agenda work streams: disease ecology, enhanced surveillance, population genomics, therapeutics, and diagnostics.

Below, we provide a brief summary of the revised evidence gaps and research opportunities by GWEP Research Agenda work stream; additional details regarding the results of the GW research gap analysis are presented in the Supplemental Materials. Within each work stream section, we also provide information related to the work stream’s scope of work, which was influenced by GWEP research needs and opportunities as identified during the research gap analysis, as well as progress and evidence to date.

### GWEP Research Agenda work streams.

The GWEP Research Agenda seeks to operationalize a broad portfolio of rigorous investigations into different aspects of GW’s multiple-host pathogen system. The programmatic research agenda was designed specifically to generate evidence that could be used to inform refined intervention design and implementation, accelerate the development and deployment of additional interventions (e.g., novel GW diagnostics, therapeutics), and guide programmatic and policy decisions. An array of applied biomedical, epidemiological, operational, and implementation research was organized within these five work streams, each of which is described below. Given it is unlikely that all research gaps and opportunities identified via the full research gap analysis (see Supplemental Materials) can be pursued in time to achieve the 2030 eradication target, initiatives with a bearing on GW transmission and an ability to be completed in a time frame that aligns with eradication goals are being prioritized currently under each work stream’s current scope of work.

### Guinea Worm Disease Ecology Work Stream.

**Figure 1.**
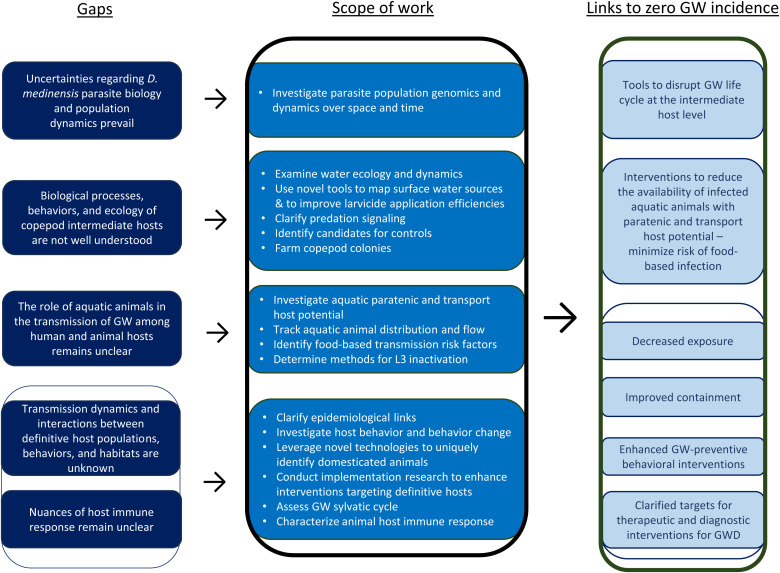
Guinea Worm (GW) Disease Ecology Work Stream: Evidence gaps, research to address gaps, and links to zero GW incidence. *D. medinensis* = *Dracunculus medinensis*; GWD = Guinea worm disease.

Disease ecology refers to interactions between the biology of pathogens and the behavior and ecology of hosts in relation to diseases that affect populations.^24^ Given that GW has a complex life cycle and the parasite is a multiple-host pathogen, GW disease ecology considers the interplay between *D. medinensis* parasite biology and population dynamics and the behavior and ecology of intermediate, paratenic, and transport hosts as well as human, domesticated animal, and wild animal definitive hosts. Figure 1 maps evidence gaps against the GW disease ecology work stream’s scope of work and anticipated outputs facilitating zero incidence of GW.

#### Research gaps and opportunities.

##### Parasite biology (D. medinensis).

The host-parasite biology and population dynamics of *D. medinensis* remain poorly characterized, but clarification regarding these details could offer insights leading to chemoprophylactic or therapeutic interventions that either prevent invasion of host tissues or disrupt parasite development. The overall understanding of *D. medinensis* pathogenesis remains at the macro level, and even at that level, evidence gaps prevail. For example, potential mechanism(s) of immune evasion, chemoattractant-modulated mating, and the elicited early immune response remain unknown. The cellular and molecular basis of biological processes, including modulators of GW parasite behavior, and immune evasion are not well understood.

##### Intermediate host biology, behavior, and habitat (copepods).

Certain biological processes, behaviors, and the ecology of copepods are not well understood; however, characterizing these phenomena could help determine organismal mechanisms to target and upgrade the nature of interventions available to address intermediate copepod host potential while improving the predictive power of environmental risk models. The sensitivity of surface water source detection could also be improved to ensure that all surface water sources in endemic and high-risk areas are being identified for intervention.

##### Aquatic paratenic and transport host biology and habitats (frogs, fish, other aquatic animals).

The role of aquatic animals in the transmission of GW among human and animal hosts remains unclear, but a better understanding of GW transmission could help further refine related interventions and minimize the risk of food-based infection. The paratenic and/or transport host potential of a few aquatic animals has been examined in laboratory and field settings^10^^,^^25^^–^^28^ but may not fully capture all aquatic paratenic or transport hosts involved in GW transmission. The factors influencing paratenic and transport host potential also remain largely uncharacterized.

##### Definitive host populations, behaviors, and habitats (humans, dogs, cats, wildlife).

Details related to GW transmission dynamics, interactions between definitive host populations, and the influence these interactions have on the distribution of GWD over space and time remain unclear but could help decrease exposure, improve containment, and enhance GW-preventive behavioral interventions. Specific modes of transmission (e.g., drinking water or consumption of aquatic animals/aquatic animal waste; species of implicated aquatic animals) still need to be elucidated and may differ by country, by location within a given country, and by definitive host. Transmission dynamics within wild animal hosts, such as baboons, and between wild animals and other definitive human and domesticated animal hosts require further investigation as well. There is still uncertainty as to whether GW infections in wild animal hosts reflect spillover events or sylvatic transmission and, if sylvatic transmission is at play, whether transmission among wildlife alone could sustain the GW life cycle. Host immune responses to GW have not been well characterized; mucosal immunity is not well understood. Immunomodulators that govern animal host immune responses have not been investigated. A better understanding of potential mechanisms of immune evasion or tolerance, such as GW-derived immunomodulators, may help guide the development of therapeutic and perhaps diagnostic interventions among animal hosts. That said, developing immunotherapies can take several years of research and development, so related investigations may not be completed in time to achieve the 2023 GW eradication certification target. Although this gap remains on the radar and some host immunity–related work may be pursued, especially in relation to animal host immunity, other research that can be completed in a timely manner will be prioritized over these investigations.

##### Scope of work under the GWEP Research Agenda.

At each step along the GW life cycle, environmental, biological, and perhaps immunological forces act against *D. medinensis*, thereby dramatically reducing the numbers of organisms that eventually complete the GW life cycle. As such, the scope of work for the GWEP Disease Ecology Work Stream includes investigations into the *D. medinensis* pathogen itself as well as the multiple hosts involved in the pathogen’s life cycle.

Investigations of *D. medinensis* parasite population dynamics over space and time comprise the current focus of the parasite biology and population dynamics research. These efforts are cross-cutting with the GW Population Genomics Work Stream.

Research at the intermediate host level includes investigations into water ecology and the influence of water ecology and larvicide treatment on copepod population dynamics, intermediate host transmission potential, copepod predation signaling (i.e., predation perception, sensory abilities resulting from hydromechanical and chemical signals), and anthropogenic and water quality factors that may influence the size and composition of copepod populations. Several of these investigations rely on the identification of candidates for possible controls and the farming of copepod colonies, efforts of which are also being expanded. Satellite images and more rigorous mapping tools are being leveraged to remotely sense water sources and more precisely target the location and timing of larvicide applications. Implementation research into novel strategies that may improve efficiencies in the measurement of the volume of water in surface water sources targeted for larvicide treatment is underway and could reduce the amount of time and human resources necessary to implement the treatments effectively.

Aquatic paratenic and transport host potential will be assessed further via field studies. The distribution, movement, and flow of potential aquatic paratenic and transport hosts over space and time will also be investigated. Potential transmission pathways that deviate from classical water-based transmission, such as food-based transmission involving aquatic paratenic or transport hosts, is under investigation via analyses of food-based risk factors of GWD. Implementation research investigating methods of L3 inactivation in aquatic animal matter will also be undertaken.

Definitive host research includes systematic examinations of case and infection investigation data to further clarify epidemiological links and transmission dynamics between and among definitive hosts as well as investigations into definitive host behavior and behavior change to enhance interventions targeting definitive hosts. Data from baboon trappings and enhanced wildlife surveillance initiatives are facilitating assessments of a GW sylvatic cycle. Research into animal host immune responses is also underway. Longitudinal research on domesticated animal hosts requires identifying novel technologies that could be used to uniquely identify animals for tracking and investigation over time. As such, different animal host identification methodologies are also being evaluated and tested. Additional implementation research efforts will include examinations into animal well-being and tethering interventions and other research to generate additional data that can inform evidence-based refinements to interventions, implementation approaches, and delivery strategies.

#### Progress and evidence to date.

Given that ongoing investigations into *D. medinensis* population dynamics are cross-cutting with the GW Population Genomics Work Stream, related progress and evidence to date are described under that work stream. Water ecology studies are currently underway to examine spatiotemporal dynamics of copepod communities, copepod transmission potential, and ecological and epidemiological processes that influence intermediate host-parasite interactions as well as the influence of predation on disease dynamics (R. Garabed and J. Lee, unpublished data).^29^^,^^30^ Investigations into the biology of Chadian copepods are aimed at discerning the factors that elicit GW predation (ingestion). Scientists are examining copepod sensory reception and GW L1 signals that trigger copepod predatory behavior toward L1s.^30^ Current implementation research initiatives are addressing intermediate host habitats and improving efficiencies for larvicide application in other ways. Novel automated and remote sensing technologies are being used when and where possible to map bodies of water and improve the identification of water sources in GW-endemic areas as well as areas otherwise at risk for propagating GW transmission and to expedite larvicide application at eligible water sources.^31^ These technologies have helped identify water sources under dense canopy and provided insights to inform actions to address potential wild animal hosts living in forested locations.^31^ In addition, an automated system that involves the use of drone boat sleds has been developed to increase the accuracy of water volume calculation and further improve efficiencies related to larvicide application at eligible water sources. A smaller, plug-and-play version is under development for use in the remaining endemic countries.

Research prompted by the detection of GW infection in dogs and cats in Chad and baboons and dogs in Ethiopia generated evidence that frogs and fish may be serving as paratenic and transport hosts, respectively.^25^^–^^28^^,^^32^^,^^33^ Studies using isotope analysis have found a direct correlation between fish diet and dog infections in Chad.^22^ However, the role that fish, amphibians, and other aquatic animals play in the transmission of GW requires further investigation, particularly in areas reporting GW infection among domesticated animals where there is limited access to aquatic animals.

A global effort is currently underway to prospectively capture field epidemiology data from investigations into analyzable datasets to facilitate the identification of person-based, animal-based, and location-based epidemiological links between and among definitive hosts.^34^ Resulting datasets will also be used to identify correlates of disease among human and animal hosts. Similar efforts to code and extract data from historical case and infection investigations are also underway to ensure the datasets are comprehensive. These data are being combined with genomic surveillance data to assist with the interpretation of genomic analytic results and to provide a more comprehensive understanding of GW epidemiology in endemic countries.

To better understand animal behavior and potential overlaps in animal host ranges, specifically at locations that may facilitate GW transmission, investigators deployed camera traps around Chadian water sources that were situated away from human habitations.^35^ The camera traps were intended to generate data on water source use by wildlife species, overlap between wildlife species and free-roaming dogs or cats, and animal behavior at the water sources more broadly. Analyses of these data are forthcoming. To document risk behaviors among domesticated cats, such as hunting aquatic animals, drinking from surface water sources, and stealing/consuming drying fish, collar cameras (i.e., crittercams) were put on cats in three villages in Chad. The results of this investigation demonstrated that on more than 80% of nights, cats were found to have demonstrated at least one potential risk behavior.^36^ Investigations into baboon behavior and GW transmission dynamics among baboon hosts are currently underway as well. Fourteen baboon troops are being monitored in Gambella, Ethiopia, through two approaches: 1) routine tracking to observe baboon behavior from a distance and 2) scheduled trapping activities to facilitate clinical exams and collect biological samples from the baboons. All baboon troops with documented evidence of GWD inhabit locations that are in relatively close proximity to each other, and most are known to share water sources and other resources with villages that have reported GWD among domesticated animal hosts. Behavioral observations of the baboon troops and an analysis of stable isotopes recovered from them indicate that these baboons eat fish and frogs, albeit rarely.^37^

### Enhanced GW Surveillance Work Stream.

**Figure 2.**
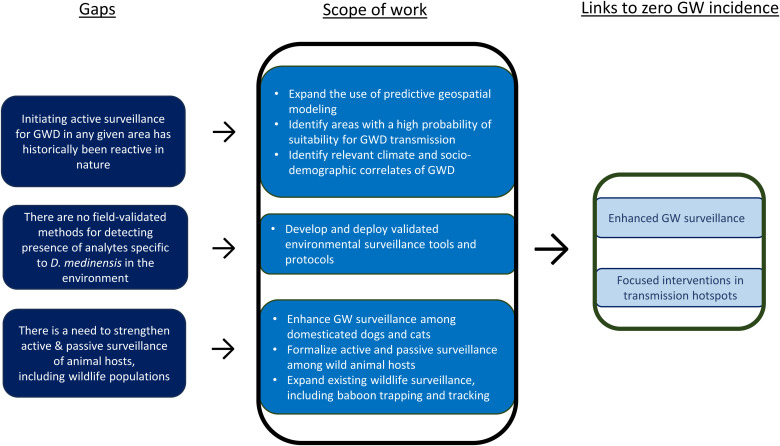
Enhanced Guinea Worm (GW) Surveillance Work Stream: Evidence gaps, research to address gaps, and links to zero GW incidence. *D. medinensis* = *Dracunculus medinensis*; GWD = Guinea worm disease.

Figure 2 maps evidence gaps against the enhanced GW Surveillance Work Stream’s scope of work and anticipated outputs facilitating zero incidence of GW.

#### Research gaps and opportunities.

Guinea worm disease surveillance infrastructure in endemic countries involves both active and passive surveillance components. Active surveillance involves a community-based approach in which community members systematically search for GWD or signs and symptoms of GWD among humans and animals on a routine basis. Initiation of active surveillance for GWD in any given area has historically been reactive in nature. Villages are placed under active surveillance when one of three criteria are met: 1) GWD is detected in the villages, 2) the villages share epidemiological links with villages where GWD has been detected, or 3) the villages demonstrate some other GWD risk factor. In contrast, passive surveillance relies on either the local health system identifying GWD or individuals reporting rumors to national GWD hotlines or GWEP staff. To enhance surveillance for GWD, national programs have integrated surveillance activities within their health systems and through large-scale outreach activities (e.g., GWD case searches during national immunization days) to leverage existing infrastructure and community engagement to promote detection and reporting of GWD. There are limitations to these approaches that could be addressed through the use of predictive modeling techniques. The expanded use of predictive geospatial modeling would facilitate the identification of areas with a high probability of suitability for GWD transmission and may help inform decisions about which areas to proactively place under active or increased surveillance for GWD based on transmission potential.

Currently, there are no field-validated methods for detecting the presence of analytes specific to *D. medinensis* in environmental media. Viable environmental assays that can detect evidence of analytes specific to *D. medinensis* in various environmental matrices, including water, aquatic animals, and perhaps aquatic animal waste,^38^ could be used to guide interventions. Such tools would be useful in endemic areas, especially those with known or suspected wildlife transmission, and during pre-certification and certification stages. Environmental surveillance tools such as these could complement existing surveillance tools and enhance surveillance in a manner unprecedented for the global GWEP.

Although many national GWEPs are implementing active, community-based GWD surveillance among domesticated animal hosts, there are still opportunities to strengthen this component of the national surveillance system. It is difficult to operationalize surveillance and infection control in wildlife. Because both wild carnivores and baboons are being detected with subcutaneous worms and/or patent infection, it is critical to have a robust and sensitive surveillance system that can also detect GWD among all wildlife species with GWD host potential.^39^

New tools, enhanced evidence, and refined strategies are needed to supplement existing GW surveillance systems and inform decision-making about when and where to implement program interventions and whether to contract or expand active community-based surveillance infrastructure. There is also a need to strengthen active and passive GW surveillance among animal hosts, including wildlife populations.

#### Scope of work under the GWEP Research Agenda.

The scope of work for the enhanced GW Surveillance Work Stream encompasses three main areas: 1) expanding spatiotemporal geospatial predictive modeling, 2) developing and validating an environmental assay that can detect analytes specific to *D. medinensis* in various environmental media, and 3) enhancing active surveillance of domesticated animals and wildlife populations.

Geospatial predictive modeling is being expanded to incorporate remotely sensed climatic and socioeconomic variables that are correlated with GWD. These variables are integrated into a modeling framework designed to predict infection risk in regions lacking surveillance data or not covered by the active surveillance system. Predictive geospatial modeling enables proactive identification of areas with GW transmission potential and may inform decisions about areas to target for case and infection searches and/or to place under active surveillance. In addition, geospatial models are being used to characterize spatially explicit habitat suitability by associating species distribution data with potential disease correlates such as climate, environmental, and sociodemographic factors.^40^^,^^41^

Environmental surveillance tools that can detect analytes specific to *D. medinensis* in environmental media in the field, such as samples of water sources and aquatic animals, are being investigated. In addition to identifying and validating effective environmental tests, field protocols that help operationalize the use and interpretation of the tests are needed so field agents can undertake appropriate actions based on the test results.

Active and passive surveillance of domesticated dogs and cats and wildlife is being enhanced in several GW-endemic countries. Wildlife surveillance systems are being enhanced to include wildlife host species already known to have harbored GW and new potential host species in case evidence of GWD among novel wild animal hosts emerges.

#### Progress and evidence to date.

Guinea Worm Eradication Program researchers have built a pipeline for implementing geospatial models based on the ecological niche modeling framework.^41^ Current GW ecological niche models reflect adaptations of previous work on lymphatic filariasis in Nigeria.^41^ Using GWD surveillance data from Chad, relevant predictor variables were incorporated into an ecological niche modeling framework to produce district-level maps that depict the environmental suitability of GW.^42^ Efforts are currently underway to extend this modeling approach to other GW-endemic countries. Resulting high-resolution risk maps may help inform decisions about where GWD case searches are conducted and whether intervention resources are deployed to areas that are suitable for GW transmission.

To address prevailing gaps related to the development and field validation of environmental surveillance tools, GWEP research partners are piloting environmental assays, researching and validating methods for sample collection and biological specimen preservation, and conducting laboratory tests on collected samples. As this is a cross-cutting activity, also see the GW Diagnostics Work Stream section for further information.

To improve the sensitivity of surveillance systems, several national GWEPs, including those in South Sudan and Ethiopia, have developed specific standard operating procedures for GW surveillance among domesticated animal hosts. Similarly, current wildlife surveillance includes both active and passive elements. As mentioned above, the 14 baboon troops that are being tracked and trapped are currently under active surveillance, with weekly reports on behavior and signs of GW among the troops observed from afar. Baboon trapping that occurs at least twice per year includes thorough clinical examinations of baboons for signs of GW and collection of biological specimens such as blood. Deceased wildlife reported by communities in Ethiopia, Chad, and South Sudan are checked for GW, and “white worms” collected from a variety of wildlife species are submitted to the Centers for Disease Control and Prevention (CDC) for laboratory testing and identification to help ensure potential wildlife hosts are detected and all GWs are accounted for.

### Guinea Worm Population Genomics Work Stream.

**Figure 3.**
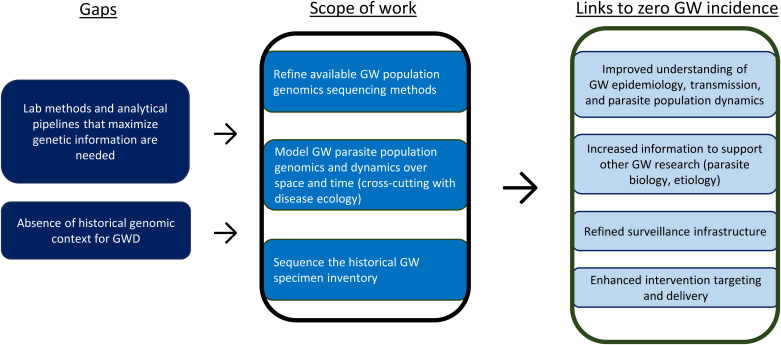
Guinea Worm (GW) Population Genomics Work Stream: Evidence gaps, research to address gaps, and links to zero GW incidence. GWD = Guinea worm disease.

Figure 3 maps evidence gaps against the GW Population Genomic Work Stream’s scope of work and anticipated outputs, facilitating zero incidence of GW.

#### Research gaps and opportunities.

The primary gaps in the application of population genomics to questions of GW population history and dynamics fall into one of two categories: 1) laboratory methodologies and analytical pipelines that do not allow the program to maximize the amount of genetic information for each individual parasite and each parasite population while also minimizing the time between sample collection and data analysis and 2) the absence of historical genomic context within which to place contemporary worms and worm populations. Addressing these two gaps requires refining available GW genomics sequencing methods and sequencing the historical GW specimen inventory. Such population genomics advancements could help clarify GW epidemiology, transmission, and parasite population dynamics while generating additional data to facilitate evidence-based decision-making regarding surveillance infrastructure and intervention targeting and delivery.

#### Scope of work for the GWEP Research Agenda.

The GW Population Genomics Work Stream addresses prevailing uncertainties regarding the genetic variability and kinship of circulating *D. medinensis* parasite populations in recently and currently endemic countries. Population genomics uses genomic data collected from all or part of an organism’s population to compare patterns of sequence variation within and between individuals. Statistical analyses of these patterns can be used to draw conclusions about GW parasite population movement and history of population growth or reduction^43^ and may help assess surveillance and intervention efforts.^44^

Two different genomic sequencing technologies are being used to understand more about the GW parasite populations and their presence across time and locations: mitochondrial DNA (mtDNA) and microsatellite sequencing. Owing to direct maternal inheritance, mtDNA genetic data extracted from worms can provide insights about the connection of current worms to ancestral lines of GW transmission and distinguish between lineages in the same region. A combination of mtDNA and microsatellite genetic data can go beyond identifying lineages to reveal evidence suggestive of kinship and recent transmission events within a single year or from one year to the next. Genetic evidence that suggests worms are siblings from the same mother can indicate a common source of infection among hosts detected in a single year, whereas genetic evidence of parent-offspring relationships can highlight lines of transmission from one year to the next.

By evaluating the amount and distribution of genetic variation in the worms detected one year, insight is gleaned into an eradication program’s surveillance and control efforts the previous year. For instance, high levels of genetic variation would suggest that programs did not detect or sufficiently contain many worms, whereas lower amounts of variation and/or clusters of highly related worms would suggest a smaller parasite population size and/or that most infections were caused by a few worms. Likewise, when GWD arises in unexpected locations (whether because there is no historical record of endemicity or because there is no reported GWD despite active surveillance), the population genomic data may be an important tool for identifying or eliminating suspected sources of infection and understanding recent local parasite population history. This is especially true as genetic data are being combined with epidemiological field data from case and infection investigations. This application of the data has already proved to be a valuable addition to epidemiological investigations of GWD outbreaks in areas without prior history of transmission,^45^ and many more targeted evaluations are underway. The geographic range over which worms maintain high relatedness could be used to infer patterns and ranges of host movement, informing biologically and epidemiologically relevant surveillance zones and clarifying the degree to which long-range host movement could pose a risk to areas without a known history of transmission.

#### Progress and evidence to date.

Early GW genomics work used “classical” methodologies such as Sanger sequencing of select mitochondrial genes (e.g., *cox1, cytB, nad3,* and *nad5*) and fragment analysis to detect length polymorphism at 24 hypervariable microsatellite loci on the nuclear genome.^46^ Concurrent to this, efforts to implement whole-genome sequencing methodologies to study GW population dynamics were also implemented.^47^ However, both the classical and whole-genome sequencing methodologies fell short of the global GWEP’s data delivery needs. Although classical protocols (which rely on specific amplification and enrichment of GW genetic targets) returned usable genetic data at a much higher rate than whole-genome sequencing, they were subject to data curation bottlenecks and returned significantly less genetic information about each worm, increasing the risk that distinct variants would be missed as eradication efforts continued to diminish parasite population sizes and, presumably, genetic variation. Conversely, although whole-genome sequencing can generate sequence data for most, if not all, of an individual worm’s genome, the sequencing protocol is not species specific and is therefore subject to interference by contaminating genomes from bacteria and host tissues, contamination that is simply unavoidable during the extraction and collection of GW from hosts. Up to 60% of GW tissue samples processed through whole-genome sequencing failed to return usable data owing to insufficient data quantity and/or data quality relative to contaminating genomes, greatly impacting the ability to gather comprehensive information about populations. To maximize the amount of genetic data per individual and the number of individuals with usable genetic data while maintaining high-throughput laboratory and analytical workflows, we adopted amplicon sequencing protocols on a next-generation sequencing platform for both mitochondrial sequence generation and microsatellite genotyping. The updated mitochondrial sequencing protocol has increased coverage of the GW mitochondrial genome almost 3-fold, resulting in significantly improved resolution of genetic variants.^44^ Likewise, massively multiplexed amplification and genotyping-by-sequencing has increased the number of microsatellite loci targeted by more than 5-fold, facilitated automated allele calling through bioinformatics pipelines, and significantly increased the detection of variants that would have otherwise gone undetected with fragment analysis methods (e.g., single-nucleotide polymorphisms within and outside of the repeat motif and insertion-deletions not associated with the microsatellite repeat).

A GWEP Genomics Working Group (GGWG), composed of members with multidisciplinary backgrounds from The Carter Center (TCC), Vassar College, the Bill and Melinda Gates Foundation’s Institute for Disease Modeling, and Emory University’s Integrated Computational Core, was formalized in 2022. The GGWG established genomics and bioinformatics pipelines to support GW genomics investigations at different scales, from the global perspective and national program viewpoints to more nuanced GWD clusters and outbreaks. A refined genomics sequencing protocol was developed, and the historical backlog of GW specimens that were collected from 12 countries during 2006–2023 has been organized and is being processed through laboratory and analytical pipelines. To expedite the production of data, mtDNA sequencing of historical samples is being outsourced to a commercial vendor. As of June 2023, a total of 15,267 GW specimens have been accounted for in the GW genomics inventory. Of these, 14,577 have been processed through or are currently in the genetic processing pipeline.

### Guinea Worm Diagnostics Work Stream.

**Figure 4.**
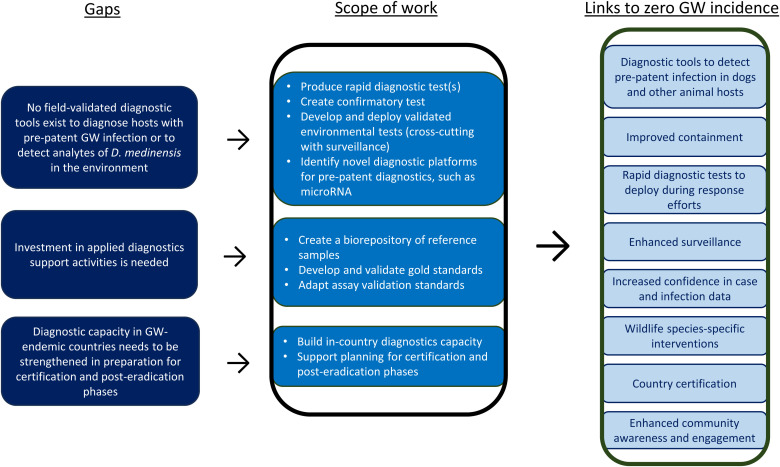
Guinea Worm (GW) Diagnostics Work Stream: Evidence gaps, research to address gaps, and links to zero GW incidence. *D. medinensis* = *Dracunculus medinensis*.

Figure 4 maps evidence gaps against the GW Diagnostics Work Stream’s scope of work and clarifies anticipated outputs facilitating zero incidence of GW.

#### Research gaps and opportunities.

No field-validated diagnostic tools exist to diagnose hosts with GW infection in the pre-patent phase (i.e., prior to worm emergence) or to detect the presence of analytes specific to *D. medinensis* in environmental media. Being able to diagnose GW infection early, after duodenal mucosal penetration and early migration, would represent a leap forward for the eradication campaign and country certification. Diagnostic modalities and platforms are needed for the early diagnosis of GWD, enhanced follow-up and containment of GW-infected hosts, and environmental surveillance. No rapid diagnostic tests (RDTs) are available to deploy during response efforts or to support country certification. Guinea worm diagnostic tools that could detect pre-patent infection or be used for environmental surveillance could increase confidence in GW case and infection data and further enhance disease surveillance.

In test development, sensitivity and specificity are evaluated and validated against known positive and negative samples of a condition from the target population and geographical location, and these known positives and negatives are termed reference samples. The status of these reference samples has been previously ascertained by a “gold standard” test. Development of reference samples is a challenge in GW infection because of the lack of a gold standard test to detect infection during the pre-patent period.

Investment in applied GW diagnostics research and development is needed, as are a biorepository of reference samples and assay validation standards. Diagnostic capacity in GW-endemic countries also needs to be strengthened in preparation for certification and post-eradication phases.

#### Scope of work for the GWEP Research Agenda.

The scope of work for the GW Diagnostics Work Stream focuses on developing a dynamic diagnostic portfolio that includes tests for early detection of pre-patent GW infection, rapid confirmation of GW infection, and detection of analytes specific to *D. medinensis* in environmental media. Multiple diagnostic tests are currently under development by different GWEP research partners focusing on diagnostics development; they include serological assays to detect IgG and IgG4 antibody responses to GWD, a nucleic acid amplification test to detect circulating cell-free *D. medinensis* DNA, an assay to detect *D. medinensis*–derived microRNAs (miRNAs), and an assay to detect *D. medinensis* DNA in water samples.^48^^–^^50^ Biorepositories of GW specimens, GW-infected copepods (that can be used to experimentally infect animal models), and clinical samples (e.g., serum, other pathogens of interest that may cross-react with GW diagnostic tests) are being expanded to improve access to samples available for developing and validating diagnostic tools. Furthermore, biorepository expansion will replenish negative control samples (e.g., negative control sera) available from GW-endemic countries. It is critical that samples used for diagnostics development be from the regions and species of interest to verify that the tests are specific to GW and not cross-reactive with other nematodes found in GW-affected areas. To strengthen and support diagnostics development and deployment now and in the future, collaborations, trainings, and other capacity-building activities are being implemented with focal points from targeted laboratories in endemic countries. Finally, because of a current lack of gold-standard for GW diagnostics, a validation method is under development to verify assertions made by all diagnostics developed so they may be used to help certify countries as free of GW.

#### Progress and evidence to date.

Despite several challenges, progress has been made on several fronts related to the development of GW diagnostic tools. A diagnostic technical advisory group was convened in 2021 to create and formalize target product profiles (TPPs) that can guide the development of in vitro tests to detect analytes specific to *D. medinensis* in animals to serve as a diagnostic test and, in environmental samples, to facilitate environmental surveillance.^51^ The TPPs were revised based on feedback provided during public consultation, and the WHO has recently published them.

The search for early GW-unique circulatory markers has been challenging for several reasons, not least of which has been the issue of cross-reactivity with other parasites that may also be infecting hosts in these endemic regions, where infection with multiple parasitic infections is common. This underscores the importance of an assay’s fit for purpose and performance characteristics, namely sensitivity and specificity, as national GWEPs make progress toward zero incidence. A promising Luminex multiplex bead serological assay based on an adult worm antigen, DUF148, has been developed.^49^ A proof-of-concept Luminex platform was optimized for dogs, detecting infections as early as 168 days (i.e., 5.6 months) after infection in two laboratory-infected dogs.^49^ Attempts are being made to adapt this platform into an ELISA or an RDT. Researchers are also investigating miRNA shed from *D. medinensis* as potential diagnostic markers of early, pre-patent GW infection.^50^ These highly stable miRNAs, shed by organisms at the host-parasite interface, are believed to have immunomodulatory properties that could be detected by polymerase chain reaction (PCR) on biofluids such as blood and serum.

A real-time PCR (qPCR) assay targeting the mitochondrial cytochrome *b* (*cytb*) gene of *D. medinensis* has been developed.^52^ This provides a qualitative diagnosis (i.e., positive or negative result) for GW infection. This method may compensate for the limited availability of well-trained microscopists and may also add objectivity to the identification of well-preserved specimens. A validation of this novel qPCR assay is currently underway using samples that were and were not properly preserved to understand how sample storage and preservation quality may influence the results.

A loop-mediated isothermal amplification (LAMP) assay has been developed to detect GW larval DNA in copepods.^38^ However, a field test platform of the LAMP assay or similar molecular test is necessary to produce a viable tool that can help determine environmental contamination by GW larvae in field settings. The clarification of a sampling strategy for the environmental media tested is also needed. A study is underway to understand the water sampling design necessary to support such a platform (R. Garabed and J. Lee, unpublished data). Ideally, diagnostics tools to detect *D. medinensis* analytes in the environment would have applications beyond water and copepods and allow for the detection of GW larvae in other environmental matrices such as aquatic animals and perhaps aquatic animal waste.

Other advancements relevant to the GW diagnostics work stream include progress made on developing biorepositories of reference samples, such as samples collected during baboon trapping sessions. A GW Diagnostics Working Group was recently formalized to facilitate stronger collaborations between research partners and to engage other subject matter experts to help accelerate the development of effective GW diagnostic tools.

### Guinea Worm Therapeutics Work Stream.

**Figure 5.**
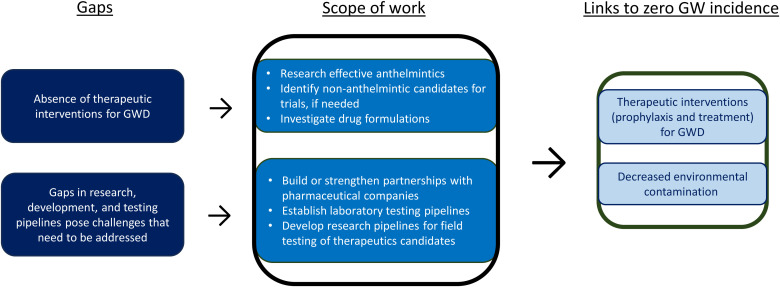
Guinea Worm (GW) Therapeutics Work Stream: Evidence gaps, research to address gaps, and links to zero GW incidence. GWD = Guinea worm disease.

Figure 5 maps evidence gaps against the GW Therapeutics Work Stream’s scope of work and anticipated outputs facilitating zero incidence of GW.

#### Research gaps and opportunities.

To date, there are no effective therapeutic options for the treatment or prevention of GWD. The only medical treatments for GWD, known since antiquity and still practiced today, involve the gradual extraction of the gravid adult female worm and management of the skin lesion to reduce associated pain and to prevent secondary infection. Therapeutic interventions for GWD could be used for chemoprophylaxis and treatment, which may also decrease the amount of GW larvae contaminating the environment. Anthelmintics (i.e., drugs or agents that act against infections caused by parasitic worms), non-anthelmintics (i.e., drugs that are not designed to act against infections caused by parasitic worms but could influence the fitness or pathogenicity of a parasitic worm), and other biotherapeutics could be evaluated, reevaluated, researched, and developed to advance therapeutic options for the treatment and prevention of GWD among dogs and perhaps cats. New drug formulations may also need to be investigated. Gaps in research, development, and testing pipelines pose challenges that need to be addressed.

#### Scope of work for the GWEP Research Agenda.

The GWEP Therapeutics Work Stream is currently focusing on evaluating a high-dose regimen of flubendazole among dogs. Depending on the results of the current study,^53^ the global GWEP may consider expanding the GW therapeutics pipeline to accelerate testing of other potential compounds in the laboratory setting, including promising existing anthelmintics and potentially existing non-anthelmintic candidates, if needed. Ideal one-time dose formulation(s) with limited cold-chain requirements and limited need for handling and restraining of animals will be investigated. Building upon experience conducting dog trials for existing therapeutics (e.g., Heartgard^®^, Advocate^®^, and flubendazole), the global GWEP is establishing a streamlined process to evaluate potential therapeutics in field settings as well.

#### Progress and evidence to date.

The scientific literature surrounding the anthelmintic treatment history for *D. medinensis* in humans and animals is rich, with most of the work done in decades past and some only available in the grey literature. More recent work has focused on laboratory testing in various animal models as well as field trials in both animals and humans. The specific anthelmintics studied to date for GW include mebendazole, cambendazole, diethylcarbamazine, methyridine, metriphonate, metronidazole, niridazole, thiabendazole, albendazole, ivermectin, and moxidectin.^54^^–^^57^ Laboratory studies of the anthelmintic flubendazole in ferrets receiving a sustained higher dose (three consecutive doses 1 month apart—two rounds) provided promising results.^58^ Ferrets treated late in the gestation of GW infection developed term gravid females with larvae exhibiting poor condition, low to no motility, and an inability to infect copepods. Consequently, a randomized double-blind trial evaluating the effectiveness of flubendazole in dogs was conducted during May 2019–October 2020 in Chad.^58^ Each dog in the study received three subcutaneous doses (one daily) of flubendazole 15 mg/kg (or placebo: Intralipid 20%; Fresenius Kabi, Uppsala, Sweden) for 3 consecutive days. This treatment protocol was repeated 6 months later and again 12 months later for a total of three series. Unfortunately, the analyses indicated that this treatment was ineffective in reducing GW infections in dogs.^58^ Additional analyses indicated that larvae collected from dogs treated with flubendazole had reduced motility, although this result was not statistically significant.^58^ However, because there was evidence suggesting that dosing was a limiting factor in achieving prevention or treatment in dogs, the study informed a second trial in dogs using a higher dose of flubendazole. A multidisciplinary panel of subject matter experts recommended evaluating the effectiveness of a single high-dose subcutaneous injection (100 mg/kg) of flubendazole to treat or prevent GW infection in dogs. This revised dosage regimen was evaluated in a second trial conducted during 2021–2023,^53^ the results of which are forthcoming.

If and when an efficacious veterinary therapeutic is discovered, it would need to be operational and safe in multiple species. In the context of domesticated animal infections in all endemic regions, operationalization is mostly concerned with a simple route of delivery and a single dose of the therapeutic. Considering the GW life cycle, field conditions, and the results from clinical trials and laboratory studies that are documented either in the peer-reviewed literature or grey literature, priority should be placed on identifying long-acting and extended-release formulations.

## DISCUSSION

Although there has been great progress on the path to eradicating GWD, recent recognitions of the importance of animal infections have triggered the need for a robust GWEP Research Agenda aimed at expanding and improving interventions available to interrupt the multiple-host GW pathogen system and accelerate progress toward eradication. This paper outlines how the global GWEP is addressing eradication endgame challenges through five research work streams: GW disease ecology, enhanced GW surveillance, GW population genomics, GW diagnostics, and GW therapeutics. We have explained how evidence gaps and research opportunities identified during a formal gap analysis informed the scope of work developed for each work stream. We also described how the GWEP Research Agenda employs a systems-informed One Health approach to investigate novel interventions and enhance existing interventions to disrupt GW’s multiple host-parasite system and interrupt disease transmission in extant endemic countries.

Disease eradication is a complicated process, and GWD eradication is unique in that it currently relies on public health interventions such as active, community-based surveillance and behavior change as opposed to vaccines and chemotherapeutic interventions. Eradication programs that target other diseases, such as polio, rely heavily on biomedical interventions.^59^ The lack of effective therapeutic interventions and sensitive and specific diagnostics that can detect pre-patent GW infections or identify GW-contaminated water sources limits the tools available to support the global GW eradication effort. In addition, the GW life cycle presents unique challenges, such as a protracted pre-patent period that precedes the emergence of a gravid female worm from a definitive host.^1^ This lengthy pre-patent period has broad implications for everything from developing laboratory models to observing the effect of interventions, which takes 10–14 months to realize. A protracted pre-patent period also requires enrolling more animals in field trials to address loss to follow-up resulting from the relatively high mortality rates of animals residing in the countries that remain endemic for GWD.

As GW burden decreases, research becomes more difficult because of small sample sizes, complex logistics, and the focus on existing cases and infections in smaller geographic areas. Local contexts, the epidemiology of the disease, and the degree of animal host involvement differ by country and region, and because eradication is the goal, all cases and infections need to be addressed in all areas in which GW transmission occurs. This is difficult owing to many external factors, including instability and conflict, that often plague remaining endemic areas.^60^

The importance of animal hosts complicates the GWD eradication endgame and shifts the focus of the GWEP Research Agenda to respond to the high burden of disease in animals and the role of animal hosts in ongoing GW transmission. Evidence generated by the GWEP Research Agenda needs to continue supporting the design and implementation of new interventions and different approaches that can enhance surveillance among definitive hosts and at the environmental level.

As progress is made across the GWEP Research Agenda work streams, new questions are likely to arise and may lead to changes in research priorities. As such, the global GWEP will continue assessing evidence gaps and the overarching scope of the GWEP Research Agenda on a routine basis, and the focus of implementation research will shift accordingly. Technical working groups that support the GWEP work streams and include partners internal and external to the global GWEP research community meet to monitor progress monthly and discuss the design and execution of the work stream’s scope of work. This infrastructure facilitates communication and collaboration among research partners and leverages the knowledge of subject matter experts both internal and external to the GWEP research community. In addition, in-person workshops are convened to address unique challenges and facilitate longer-term planning for specific technical working groups. At strategic time points, technical working groups meet with relevant staff from national GWEPs and Ministries of Health to gain a better understanding of program priorities and discuss ideas for how work pursued under the work stream can assist the GWEPs in enhancing their programmatic efforts and making evidence-based decisions. Emerging evidence is also presented by various GWEP research partners annually during the global GWEP Review Meeting, so all national programs and research partners are made aware of advancements of the larger GWEP portfolio and can discuss applications of the research for their own programs. Throughout the year, individual meetings are convened between research partners and national GWEPs to share research results and to discuss plans for programmatic and policy applications. Together, these convenings facilitate collaboration and cooperation between the various GWEP stakeholders, research partners, national programs, and TCC staff, which helps maximize the research agenda and applications that can accelerate eradication efforts.

There are strengths and limitations of the methods used to conduct the research gap analysis and inform the scope of work for the different research work streams. The “call to action” convening in 2018 involved stakeholders from TCC, the WHO, the CDC, the Bill & Melinda Gates Foundation, and several global research partners and organizations. Subject matter experts both internal and external to the global GWEP were engaged to provide inputs regarding evidence gaps and research opportunities, which expanded on earlier research efforts. Updates to the GWEP Research Agenda that occurred in 2020 and 2023 involved multiple partners as well. Despite this broad range of perspectives, a more rigorous approach, such as the Delphi method,^61^ was not used to conduct the GW research gap analysis and formulate the GWEP Research Agenda work streams, and all stakeholders were not engaged at each step. Engagement of researchers and staff from government line ministries in endemic countries could have been pursued in a more focused and systematic manner as well.

## CONCLUSION

An unexpected, abrupt shift in the epidemiology of GWD has been observed over the last decade, with a higher proportion of GWD being detected among animal hosts relative to human hosts. In response to this shift and other eradication endgame challenges, the global GWEP has developed a comprehensive research agenda. This paper provides a rationale for and details related to the GWEP Research Agenda, which leverages findings from a gap analysis to address GW’s multiple-host pathogen system and to formalize its five work streams. The approach to and content of the GWEP Research Agenda is mission critical: It allows stakeholders to keep a finger on the pulse of emerging GWD transmission dynamics and guides the development and refinement of novel interventions across different work streams to accelerate zero incidence of GWD among all hosts and support the pursuit of the 2030 GWD eradication target.

## Supplemental Materials

10.4269/ajtmh.23-0889 Supplemental Materials
