# Supplementary material for: Slaying the Serpent: A Research Agenda to Expand Intervention Development and Accelerate Guinea Worm Eradication Efforts
Source: Am J Trop Med Hyg. 2024 Jul 9;111(3 Suppl):12–25. doi: 10.4269/ajtmh.23-0889 (PMC11376128; doi:10.4269/ajtmh.23-0889)
Supplement: Supplemental Materials [file tpmd230889.SD1.pdf]

SUPPLEMENTAL MATERIALS

Slaying the serpent: A research agenda to expand intervention development and accelerate Guinea worm eradication efforts

Maryann G. Delea[1], Alexandra Sack[1], Obiora A. Eneanya[1], Elizabeth Thiele[2], Sharon L. Roy[3], Dieudonne Sankara[4], Kashef Ijaz[1], Donald R. Hopkins[1], Adam J. Weiss[1*]

DETAILED RESULTS FROM THE GUINEA WORM RESEARCH GAP ANALYSIS, INCLUDING RESEARCH OPPORTUNITIES

Parasite biology (*D. medinensis*)

The following were identified as evidence gaps that may be addressed through future research into the cellular and molecular basis of biological processes and immune modulation:

- ***Understanding tissue invasion*.** Elucidating the mucosal invasion and penetration process (e.g., key organelles, secretions, or modulators) would highlight potential opportunities to prevent invasion.
- ***Clarifying tissue migration.*** Gaining insight into the migration behavior of male and female larvae independently, pre- and post-mating (adult), particularly modulators and receptors that govern this behavior could highlight critical points to exploit to halt migration through blockers, analogs, or immunotherapy.
- ***Researching metabolism*.** Investigating energy sources used by migrating GW larvae and adults once the GI tract and anus obliterate, particularly in sperm-producing males and gravid females with high energy and iron demands, may reveal critical mechanisms relevant to this chokepoint, such as a novel endosymbiont and corresponding antimicrobial(s).
- ***Investigating molting inhibitors*.** Researching or screening compounds that could disrupt or halt molting should be considered as an area for future research.

Intermediate host biology, behavior, and habitat (*Copepods*)

Related research opportunities include:

- ***Examining water ecology, dynamics.*** Characterizing the natural spatial and temporal dynamics of copepod communities and their transmission potential may generate evidence to inform the development and design of interventions to interrupt GW transmission at the intermediate host level. Evaluating the ecological and epidemiological consequences of aquatic animal predators on copepods could help clarify how to address the opposing roles of aquatic animals as biocontrol agents versus paratenic or transport hosts. Assessing the impact of larvicide application on copepod populations and the factors that influence the effectiveness of water source treatments could help inform implementation refinements regarding when, where, and how larvicide application takes place.
- ***Leveraging novel tools to map surface water sources, to improve larvicide application efficiencies.*** Locating every surface water source in GW-endemic areas and those at risk for GW transmission is important to ensure all water sources with transmission potential receive larvicide applications in a routine and timely manner. In some densely forested areas where transmission may occur, it is difficult for field teams to identify all eligible surface water sources for treatment. Novel tools to remotely sense and map surface water sources could help close this gap. Calculating the volume of water in stagnant surface water sources, which dictates the amount of larvicide applied to the water

sources, is a cumbersome, often time-consuming process. Devices that facilitate automated or semi-automated calculations could help improve larvicide application efficiencies.

- ***Clarifying predation signaling.*** Investigating the biology of susceptible copepods may clarify chemical or mechanical interference mechanisms that could be exploited to disrupt signaling that allows the detection of L1s or triggers copepod predation on GW L1s.
- ***Investigating insect growth regulators (IGR).*** Investigating IGRs as possible alternatives to the organophosphate larvicide temephos could provide additional options for the control of copepods. IGRs are safe, effective, and demonstrate low toxicity amongst mammalians. Unlike organophosphate larvicides, IGRs may intervene on multiple stages of the GW life cycle, such as molting, sterility, fecundity, embryogenesis, and egg mortality.
- ***Identifying candidates for homologous biological control*.** In addition to laboratory-based studies, investigating copepod population dynamics in areas with no or low incidence of GWD to examine the predominant species or phenotypes that may be refractory to infection could help identify homologous candidates for biological control.
- ***Farming of copepod colonies*.** Determining best practices and standard operating procedures (SOPs) for farming susceptible copepod populations would facilitate sufficient access to intermediate host models for use in laboratory studies. SOPs need to be developed to standardize many aspects of copepod-related research, including small-scale farming and harvesting practices, manipulating copepods, and streamlining the importation of wild-caught copepods from GW-endemic countries to periodically replenish laboratory-based colonies.

Aquatic paratenic and transport host biology and habitats (fish, frogs, other aquatic animals)

The following areas of investigation may be warranted:

- ***Investigating aquatic paratenic and transport host potential*.** Conducting in-depth environmental studies on aquatic paratenic and transport hosts, and elucidating species of concern as well as the age, size, and seasonality of potential aquatic animal hosts could further clarify aquatic paratenic and transport host potential.
- ***Understanding aquatic animal distribution and flow*.** Tracking the distribution and flow of aquatic animals in the environment, enumerating large collective and mass fishing points, and clarifying aquatic animal distribution pathways may help identify common origins and sources of food-based GW transmission that could be targeted for enhanced intervention.
- ***Identifying risk factors of food-based GW transmission*.** Determining risk factors of food-based GW transmission (e.g., methods used to capture the aquatic animals, consumption patterns of raw and undercooked aquatic animals [entrails vs. muscle mass or other flesh], and methods of food preparation [e.g., sun-drying, smoking, boiling]) could help refine behavior change interventions to further minimize risk of infection.
- ***Determining methods for L3 inactivation*.** Exploring context-appropriate food preparation methods that could be used to inactivate L3s and minimize the risk of GW transmission via the consumption of fish, frogs, and other aquatic animals by humans and animals. A better understanding of effective inactivation methods could also inform the refinement of behavior change messaging.

Definitive host populations, behaviors, and habitats (humans, dogs, cats, wildlife)

The following gaps and opportunities were identified:

- ***Clarifying epidemiological links among and between definitive hosts*.** Leveraging field investigation data may help clarify host-specific and location-specific epidemiological links between and among cases and infections, which may reveal common origins of infection (i.e., pinpoint focal points of transmission) and shared sources of infection among hosts. Combining field investigation data with

molecular epidemiological data from microsatellite genomics analyses could paint a more complete picture of GW epidemiology in extant endemic countries.

- ***Understanding host behavior and behavior change.*** Unpacking and documenting the array of contextual, technological, and psychosocial factors that influence human and animal behavior and the uptake or rejection of GW-preventive practices may help clarify certain aspects of GW transmission while also shedding light on factors that need to be addressed to prevent exposure and improve containment. Related data could be used to help refine the design of behavioral interventions and behavior change communication messaging.
- ***Leveraging novel technologies to uniquely identify domesticated animals.*** Evaluating and identifying novel technologies that could be used to uniquely identify animals and link them to their owners and households (e.g., barcodes incorporated into identification cards or animal collars, radio frequency identification [RFID] tags, or other biometrics [iris scanning]) could enhance surveillance, improve the tracking of animals over time, and allow for a more accurate census of the domesticated animal population at risk for GWD.
- ***Using evidence from implementation research to enhance interventions.*** Conducting implementation research could generate data used to inform the refinement of current intervention implementation approaches and facilitate the systematic uptake of clinical research findings and other evidence-based practices into routine programmatic and policy action. The following opportunities were identified for future implementation research:
    - ***Animal health.*** Designing and implementing evidence-based interventions to improve, monitor, and maintain animal health and welfare may help prevent medical conditions resulting from or exacerbated by prolonged tethering.
    - ***Multi-pronged, multi-sectoral population management strategy for dogs & cats.*** Researching and developing a combination of surgical and non-surgical methodologies for population management of dogs and cats may halt and stabilize the expanding growth of these animal populations. Formative research is required to generate data that could inform community buy-in on population management and development of related communication messages while also clarifying options to address re-homing and re-population.
    - ***Compliance and accountability program to support proactive tethering of dogs & cats.*** Exploring options for strategies that could be put in place to achieve at least 80% compliance with animal tethering intervention criteria (i.e., shelter, food, exercise, safe water, sanitation and hygiene for animals), such as active engagement and discussion with affected communities, could help improve the success of prolonged tethering interventions. The strategy should specify accountability indicators, a data collection and analysis plan, and corrective actions. Trialing options for tamper-proof collars, chains, harnesses, or alternatives for dogs and cats may increase compliance with proactive tethering interventions.
- ***Assessing the sylvatic cycle.*** Examining spillover range and the potential for GW to sustain a sylvatic cycle could help clarify the role of wildlife in GW transmission, including searching for and identifying additional definitive host species.
- ***Characterizing the immune response in animal hosts.*** Investigating the cellular and humoral immune response at the mucosal level and researching immunomodulators, cytokines, and signaling involved in immune evasion and interference with the Th2 response in animal hosts may help inform therapeutic and potentially diagnostic tool development.

Surveillance

The following opportunities may be leveraged to further enhance GW surveillance:

- ***Expanding spatiotemporal analyses.*** Executing additional spatiotemporal analyses could help predict the probability of GWD emerging in areas under passive surveillance. Examining additional modelling applications, such as geospatial risk mapping, that incorporate known and hypothesized climatic, environmental, and sociodemographic correlates of disease may help identify potential hot spots for transmission.
- ***Developing environmental surveillance tools.*** Producing and validating technologies and methodologies to assess environmental contamination with GW could offer tools for environmental surveillance (cross-cutting with diagnostics-related gaps and opportunities related to interrupting transmission and post-eradication surveillance).
- ***Enhancing active and passive surveillance of animal hosts, especially wildlife populations.*** Enhancing active and passive surveillance among domesticated dogs and cats could yield more sensitive GW surveillance systems. Formalizing active and passive surveillance infrastructure for wildlife populations could generate data that may help clarify disease ecology in wildlife (e.g., transmission routes; epidemiological links to human hosts, other animal hosts, or GW-affected locations).

Population genomics

The following areas reflect opportunities for future research:

- ***Optimizing sequencing methods.*** Adapting and refining available sequencing methods could capitalize on massively parallel sequencing technologies to enhance the breadth and depth of both genome and population coverage, improve the granularity of genetic data, expedite results, and maximize the application of genomics analytics to more efficiently partner with programs and program evaluations.
- ***Sequencing the historical GW specimen inventory.*** Conducting genomic analyses on the historical repository of worm specimens, which comprises thousands of specimens dating back to 2006, is necessary to glean any available information about historically circulating parasite populations and the historical distribution of genetic variation, as well as to enhance any available capacity for longitudinal data collection to better understand long-term genetic dynamics of GW populations.
- ***Modeling GW parasite population genomics.*** Modeling GW parasite population genomics and changes in the genetic variability of GW parasite populations over space and time could help determine whether cases and infections of GWD are going undetected or uncontained and whether program interventions are effective in reducing the genetic pool of circulating GW parasite populations, which would be expected in the lead up to disease eradication.

Diagnostics

The following were determined to be areas for research and development for GW diagnostics:

- ***Producing rapid diagnostic test(s).*** Developing veterinary rapid diagnostic tests (RDTs) could assist with the screening of dogs and cats to detect pre-patent infection. Considerations should be put in place for adapting an RDT for baboon use. Genomics, proteomics, and other similar technologies may be used to identify targets for diagnostic development.
- ***Creating a confirmatory diagnostic test.*** Developing a highly specific confirmatory test in parallel to the RDT screening tool could facilitate the production and use of the confirmatory test shortly after the rapid screening test is operationalized. Both tools are needed to inform intervention decision-making and will likely support the generation of evidence required for certification by the ICCDE.

- ***Developing environmental surveillance tools.*** Producing and validating technologies and methodologies to assess environmental contamination could offer tools for environmental surveillance (cross-cutting with surveillance-related gaps and opportunities).
- ***Identifying novel diagnostic platforms.*** Exploring and supporting the development of novel diagnostic platforms outside the realm of traditional diagnostic modalities; for example, clusters of regularly interspaced short palindromic repeats (CRISPR) and microRNA (miRNA). Identifying and characterizing immunogenic epitopes in L3 sand L4s that could be applied to the refinement of serological testing applications could facilitate early detection of GW exposure.
- ***Creating a biorepository of reference samples.*** Conceptualizing, creating, and instituting a biorepository of characterized reference materials could facilitate access to reference samples required to execute the research that could facilitate the timely generation of novel evidence and tools to bolster eradication efforts. Enhancing parasitic sample collection and preservation techniques could improve the quality of testing and boost molecular identification.
- ***Developing & validating gold standards.*** Creating serologic and molecular gold standard tests is necessary to establish baselines and efficiently optimize and validate novel diagnostic assays that are in development.
- ***Adapting assay validation standards.*** Developing widely recognized guidelines endorsed by nationally/internationally recognized entities could ensure that any diagnostic test developed is subject to validation standards that conform to widely adopted standards.
- ***Strengthening capacity.*** Developing subject matter expertise within country and regional reference laboratories could support the sustainability of diagnostic expertise. Establishing a program to train and build the next generation of diagnostics subject-matter experts could address a constricting workforce and support the demands of country certification and post-eradication activities.

Therapeutics

The following avenues have been identified as opportunities to address the treatment gap for GWD in animal hosts:

- ***Researching effective anthelmintics.*** Investigating different dosage regimens or formulations of existing anthelmintics, such as flubendazole, (e.g., higher doses, improved long-acting/extended release formulations, delivery mechanisms) could help determine whether they are effective for treatment and prevention (i.e., reduced fecundity, viability, and motility) of GWD in dog hosts.
- ***Identifying effective non-anthelmintics.*** Researching and screening commercially available biotherapeutics and modulators of the immune response (e.g., cytokine-blockers, growth factors, antibodies, other regulatory peptides and proteins, and antibiotic) may led to drug discovery for effective therapeutics for GWD. Screening of commercially available compounds, based on the results of the cytokine profiling, could allow for identifying therapeutics that may facilitate a more robust immune response to *D. medinensis.*
- ***Investigating new drugs and formulations.*** Evaluating several newer generation and off-label products (e.g., emodepside, extended-release moxidectin, derquantel, and monepantel) may be tested alone or as a potentially synergistic drugs when used in combination with therapeutics to treat or prevent GWD in domesticated dogs and cats. Using genomics, proteomics, and other similar technologies to mine for potential therapeutic compounds may also accelerate drug discovery.
- ***Building partnerships with pharmaceutical companies.*** Developing partnerships with veterinary pharmaceutical companies may advance research, exploration, and development of other possible therapeutics that may be effective at treating or preventing GWD in animal hosts.
- ***Establishing laboratory platforms.*** Testing through in vitro culture and animal models can expedite safety and efficacy testing pipelines for compounds. Optimizing culture systems, cryopreservation,

an in vivo storage of GW larvae at various larval stages would be key to successful testing pipelines. These steps could help overcome the protracted pre-patent period of GW.

- ***Developing research pipelines for field testing.*** Developing research pipelines could help streamline laboratory trial to field trial transitions while leveraging lessons learnt and best practices from previous endeavors.
